# Impact of emergency department arrival time on door-to-needle time in patients with acute stroke

**DOI:** 10.3389/fneur.2023.1126472

**Published:** 2023-03-30

**Authors:** Latha Ganti, Amber Mirajkar, Paul Banerjee, Tej Stead, Andrew Hanna, Joshua Tsau, Mohammed Khan, Ankur Garg

**Affiliations:** ^1^Department of Neurology, University of Central Florida College of Medicine, Orlando, FL, United States; ^2^Department of Emergency Medicine, University of Central Florida College of Medicine, Orlando, FL, United States; ^3^Polk County Fire Rescue, Bartow, FL, United States; ^4^HCA Florida Osceola Hospital, Kissimmee, FL, United States; ^5^Department of Mathematics and Physics, Brown University, Providence, RI, United States; ^6^Division of Pediatric Emergency Medicine, University of Florida, Jacksonville, FL, United States; ^7^Department of Emergency Medicine, UT San Antonio, San Antonio, TX, United States

**Keywords:** intravenous thrombolysis, door-to- puncture time, acute ischemic stroke, stroke system of care, emergency medicine

## Abstract

**Background:**

This study aimed to identify which emergency department (ED) factors impact door-to-needle (DTN) time in acute stroke patients eligible for intravenous thrombolysis. The purpose of analyzing emergency department factors is to determine whether any modifiable factors could shorten the time to thrombolytics, thereby increasing the odds of improved clinical outcomes.

**Methods:**

This was a prospective observational quality registry study that included all patients that received alteplase for stroke. These data are our hospital data from the national Get With The Guidelines Registry. The Get With The Guidelines^®^ Stroke Registry is a hospital-based program focused on improving care for patients diagnosed with a stroke. The program has over five million patients, and hospitals can access their own program data. The registry promotes the use of and adherence to scientific treatment guidelines to improve patient outcomes. The time of patient arrival to the ED was captured *via* the timestamp in the electronic health record. Arriving between Friday 6 p.m. and Monday 6 a.m. was classified as “weekend,” regardless of the time of arrival. Time to CT, time-to-lab, and presence of a dedicated stroke team were also recorded. Emergency medical services (EMS) run sheets were used to verify arrival *via* ambulance.

**Results:**

Forty-nine percent of the cohort presented during the day shift, 24% during the night shift, and 27% on the weekend. A total of 85% were brought by EMS, and 15% of patients were walk-ins. The median DTN time during the day shift was 37 min (IQR 26–51, range 10–117). The median DTN time during the night shift was 59 min (IQR 39–89, range 34–195). When a dedicated stroke team was present, the median DTN time was 36 min, compared to 51 min when they were not present. The median door-to-CT time was 24 min (IQR 18–31 min). On univariate analyses, arriving during the night shift (*P* < 0.0001), arriving as a walk-in (*P* = 0.0080), and longer time-to-CT (*P* < 0.0001) were all associated with longer DTN time. Conversely, the presence of a dedicated stroke team was associated with a significantly shorter DTN time (*P* < 0.0001).

**Conclusion:**

Factors that contribute most to a delay in DTN time include arrival during the night shift, lack of a dedicated stroke team, longer time-to-CT read, and arrival as a walk-in. All of these are addressable factors from an operational standpoint and should be considered when performing quality improvement of hospital protocols.

## Introduction

It is common knowledge that, in acute ischemic stroke (AIS), expeditious thrombolysis with tissue plasminogen activator (tPA) has markedly positive outcomes. Shorter time to thrombolytics—often referred to as door-to-needle (DTN) time—not only reduces mortality and symptomatic intracranial hemorrhage, but also results in higher rates of independent ambulation at discharge, discharge to home, and better functional outcomes at 3 months (1). While thrombolytics are approved to be given in the 4.5 h following the onset of stroke symptoms (with several exceptions), there is a push to give them as soon as possible, with a common mantra being “time is brain.” Giving thrombolytics as soon as 45 min following presentation to the emergency department (ED) results in improved all-cause mortality or all-cause re-admission at 1 year, although it did not impact re-admission for stroke or other cardiovascular diseases (1).

Furthermore, the updated stroke guidelines by the American Heart Association (AHA) and the American Stroke Association (ASA) (2) reflect this drive to administer thrombolytics within an hour of patient arrival, supported by additional medical associations, such as the American College of Cardiology (3). However, studies have shown that only 30%−50% of American patients with stroke have been treated within the 60-min window (4, 5). These findings motivate intensive efforts to accelerate hospital presentation and thrombolytic treatment in patients with AIS.

The 2018 AHA/ASA Guidelines for the Early Management of Patients with Acute Ischemic Stroke also addressed the pre-hospital approach to patients demonstrating stroke-like symptoms. The use of emergency medical services (EMS) has been associated with earlier arrival to the ED (<3 h), quicker evaluation, shorter door-to-imaging time (< 25 min), more rapid administration of thrombolytics (<60 min), and more eligible patients administered thrombolytics (6). Given the time-sensitive nature of AIS, the public has been encouraged to call EMS if they experience stroke-like symptoms; however, only 60% of patients with stroke are brought by EMS (7). One of the largest benefits of EMS transporting the patient is the advance notice provided, allowing the ED to assemble the hospital stroke team for rapid evaluation and care. Furthermore, advance notice allows the Radiology department to allocate a CT scanner for the patient with stroke, minimizing time wasted if another patient is being scanned. Streamlined protocols across providers and specialties have shown a reduction in DTN time, regardless of the time of day or academic vs. community center (8, 9).

Many of these studies, however, were performed in Canada or in large established academic U.S. hospitals (6–9). There is a paucity of studies at community hospitals in the American southeast, especially in Florida. A major effort to remedy this, from 2010 to 2015, was the National Institute of Neurologic Disorders and Stroke (NINDS)-funded Florida-Puerto Rico (FL-PR) Collaboration to Reduce Stroke Disparities (CReSD) Study. The main findings were that the achievement of DTN time ≤60 min and DTN time ≤45 min was highest in South Florida (50%, 23%), while lowest in West and Central Florida (28%, 11%) (5). Thus, our study in Central Florida aimed to identify whether the time of day when the patient presented to our ED and whether they arrived *via* ambulance impacted the DTN time.

## Methods

This is a prospective observational quality registry study that included all patients at our institution who received alteplase in the ED. Our institution sees over 80,000 patient visits per year, has a comprehensive stroke center, and is home to both neurology and emergency medicine residency programs. Of the 80,000 ED visits, ~500 are for acute ischemic stroke, and we deliver acute thrombolysis for ~25%. The current study consisted of consecutive patients over a 12-month period that presented as stroke alerts and received thrombolytic therapy. The University Institutional Review Board gave an exempt determination for this study #SBE-1814176.

Our acute stroke protocol begins with notification from emergency medical services (EMS) personnel for patients transported *via* ambulance. Our prehospital protocol to screen for stroke includes the Los Angeles Motor Score (LAMS), baseline functional status, and current anticoagulant (5). Immediately upon notification, a “stroke alert” is announced on the overhead pager, and stroke team members are individually notified on their hospital cellular phones. The stroke team consists of a neurology resident physician, a neurology advanced registered nurse practitioner, the CT and lab technicians, the ED pharmacist, and the ED physicians. During the period of this study, our institution did not have 24/7 coverage by the stroke team. The team is, thus, able to mobilize before patient arrival when prehospital notification is received. Upon arrival, the patient is transported to a designated “stroke bed,” which automatically weighs the patient. The thrombolytic dosing is calculated based on this weight and is readied in case the patient would qualify for thrombolysis. The patient's blood is obtained for laboratory analysis en route to the CT scanner, and thrombolytics are administered in the CT scanner room itself if the patent is a candidate.

The DTN time along with other stroke metrics was captured in the Get with the Guidelines^®^-Stroke (GWTG) registry, to which our institution belongs. GWTG-Stroke is the American Heart Association's (AHA) collaborative performance improvement program, demonstrated to improve adherence to evidence-based care of patients hospitalized with stroke (6). Hospital data are entered into GWTG by trained and monitored abstractors using explicit protocols, precisely defined variables, and standardized abstraction instruments, as delineated by the AHA. Missing, conflicting, and ambiguous chart elements are coded per AHA GWTG instructions. Abstractors are blinded to any study hypotheses, as they are data entry personnel who work for the stroke service and are not part of the research team.

The time of patient arrival to the ED was captured *via* the timestamp in the electronic health record (EHR) and categorized as either being during the “day shift” (6 a.m.−6 p.m.) or the “night shift” (6 p.m.−6 a.m.). Arriving between Friday 6 p.m. and Monday 6 a.m. was classified as “weekend,” regardless of the time of arrival. This classification is based on the shift times of the emergency department physicians as well as the stroke team. The mode of arrival was classified as arrival *via* EMS vs. walk/drove in. Our logic in selecting these two factors is that they are potentially ones that we would be able to influence.

## Results

The cohort consisted of 107 patients, 44% of whom were women. The racial distribution was 44% white, 35% Hispanic, 16% black, and 5% mixed race. The median age was 67 with an interquartile range of 55–79 years. Twenty-eight percent had a history of a prior stroke, while 36% had a history of diabetes. The median NIHSS at admission was 10 with an interquartile range of 5–17 and a range from 1 to 34. The median NIHSS at discharge was 2 with an interquartile range of 0–6 and a range from 0 to 34. The median door-to-CT time was 24 min (IQR 18–31 min). The median door-to-lab result time was 41.5 min (IQR 32–58). Twenty-four percent underwent thrombectomy in addition to thrombolysis.

Forty-nine percent of the cohort presented during the day shift, 24% during the night shift, and 27% on the weekend. A total of 85% were brought by EMS, and 15% of patients were walk-ins. The median DTN time during the day shift was 37 min (IQR 26–51, range 10–117). The median DTN time during the night shift was 59 min (IQR 39–89, range 34–195). When a dedicated stroke team was on duty, the median DTN time was 36 min compared to 51 min when they were not present. Table 1 presents the cohort demographics categorized by arrival during the day or night shift.

**Table 1:** Cohort demographics.

	**Day shift (*n* = 82)**	**Night shift (*n* = 25)**
Age	Median 67 (IQR 57–81 years)	Median 59 (IQR 44–70 years)
Sex	49% female (*n* = 41)	32% female
History of prior stroke	27% (*n* = 22)	32% (*n* = 8)
History of diabetes	41% (*n* = 34)	12% (*n* = 3)
Underwent thrombectomy	29% (*n* = 24)	16% (*n* = 4)
Race	46% white, 35% Hispanic, 13% black, 6% mixed	36% white, 32% Hispanic, 24% black, 8% mixed
Time to CT	Median = 22 (IQR 18–28 min)	Median = 30 (IQR 19–42 min)
Time to Lab	Median = 41 (IQR 32–56 min)	Median = 42 (IQR 34–58 min)
NIHSS in the ED	Median = 10 (IQR 6–17)	Median = 7.5 (IQR 2.5–15 min)
Arrival *via* walk-in	6%	28%

CT, computed tomography; NIHSS, National Institutes of Health Stroke Scale; ED, emergency department; IQR, interquartile range; min, minutes.

Univariate factors associated with increased DTN time included: arriving during the night shift (*P* < 0.0001) not arriving *via* EMS (*P* = 0.0080), and during the absence of a dedicated stroke team (*P* < 0.0001). Time to CT was also significantly associated with DTN time (*P* < 0.0001). Interestingly, time-to-lab was not statistically significant. During the day shift, the median door-to-CT time was 22 min (IQR 18–29, range 5–129) compared to 31.5 min (IQR 18–42, range 13–82) during the night shift. Arrival *via* EMS occurred in 75% of cases during the night shift, but in 88% of cases during the day shift. The time-to-CT was also significantly different for those who did and did not arrive *via* EMS. The median time-to-CT for patients arriving *via* EMS was 21.5 min (IQR 17–28, range 5–129 min). By contrast, the median DTN time for patients not arriving by EMS was 37 min (IQR 25.5–50, range 19–62 min).

None of the other factors, including age, sex, history of diabetes or prior stroke, or NIHSS in the ED, were significant. In a multivariate model that included all of these factors, the same univariate factors remained statistically significant.

Of these univariate results, the ones that were most interesting to our team were the time of patient arrival (day vs. night shift and weekday vs. weekend shift) and mode of arrival, as these are operational factors that we could potentially address. We performed a two-way analysis of variance (ANOVA), which is a whole-model test that determines whether at least one pair of means is significantly different from each other. After rejecting the null hypothesis (*P* < 0.0001), we followed up with Tukey's multiple-comparison procedure, a conservative test that adjusts for multiple comparisons, and found that night shift patients had a significantly longer DTN time than day shift patients (*P* < 0.0001) and weekend patients (*P* = 0.0272).

To determine the effect sizes, we performed a least-squares linear regression model. This model yields a line that makes the vertical distance from the data points to the regression line as small as possible. It is referred to as a “least squares” because the best line of fit is one that minimizes the variance (the sum of squares of the errors). The predicted DTN time (*R*^2^ = 0.281) was determined by the following approximate formula: 48.5 min + 18.1 min (if the patient is a walk-in) + the value for shift time. Values for shift time were as follows: −16.0 min if the day shift, +15.3 min if the night shift, and +0.6 min if the weekend shift. Both the shift type (*P* < 0.0001) and arrival by walk-in (*P* = 0.0095) were statistically significant. As the weekend shift contains both day and night shifts, the effect size is close to zero for weekend patients, as the changes in DTN time by shift type cancel each other out.

As the weekend shift seems to be a flawed grouping of data (because it groups both night and day shifts together and the main difference exists between these two groups), we repeated our linear regression model with the exclusion of the weekend patients (*n* = 74, *P*-value through ANOVA < 0.0001, *R*^2^ = 0.36). We found that both arrivals during the night shift (*P* < 0.0001) and arrival by walk-in (*P* = 0.0047) were significantly associated with higher DTN times. The predictive formula for DTN time, in this case, was: 32.2 min, plus 21.5 min if arriving *via* walk-in, plus 30.8 min if arriving *via* night shift.

Our second linear regression model, which excluded patients who arrived on the weekend, was found to have a much better coefficient of determination in predicting DTN times. This is not because weekend patients are unique, but rather a result of the patient grouping in our database. The effect of weekend vs. weekday is dwarfed by the effect of day vs. night shift, which were initially both included as “weekend patients.”

Six patients in our cohort suffered a post-tPA hemorrhage. While not statistically significant, the median DTN time for those with a hemorrhage was 44 min (IQR 29–74) vs. 40.5 min (IQR 30–58) in those who did not have a post-tPA hemorrhage.

We reviewed each of the cases in which there was a prolonged DTN time. The majority of these cases had to do with not immediately recognizing that the presentation was an acute stroke. A disproportionate number of these cases occurred during the night shift (Table 2).

**Table 2:** Reasons for delay in door-to-needle (DTN) time.

**Reason for delay in DTN time**
Patient was brought in by EMS as a drug overdose
Transfer from free-standing ED and delay in getting INR
Patient unstable
Chest pain and concern for aortic dissection, needed to wait for chest CTA
Patient improved then worsened
Atypical stroke symptoms; needed intubation and repeat CT to rule out bleeding due to worsening neuro symptoms
Chest pain and need to rule out aortic dissection
Neurology delay in calling back
Patient with dementia, unable to give a reliable time of onset
Elevated blood pressure requiring control prior to tPA
Patient initially presented as a drug overdose
Patient presented with a complaint of chest pain initially
Unclear about anticoagulant use (patient with a history of atrial fibrillation)
Patient with waxing/waning symptoms

EMS, emergency medical services; ED, emergency department; INR, international normalized ratio; CTA, computed chest tomography; CT, computed tomography; tPA, tissue plasminogen activator.

## Discussion

Our study sought to ascertain the factors influencing DTN time at our mid-sized academic community ED in the Southeastern United States. The data show that a patient's DTN time was longer if they arrived as a walk-in or if they arrived during the night shift.

There are several possible reasons why our data showed a statistically significantly higher DTN time at night, despite the stroke protocol for all times of the day being the same.

First, the night shift is inconsistently staffed, with many nights having no dedicated stroke team covering, so it falls to the ED team to see all patients including patients with stroke. ED resident staffing is also decreased at night, with fewer senior residents, and there is usually a team consisting of one senior resident and two interns. With fewer helping hands, each individual has more tasks, and all of these tasks can slow down the process.

In the National Institute of Neurologic Disorders and Stroke (NINDS)-funded Florida-Puerto Rico (FL-PR) Collaboration to Reduce Stroke Disparities (CReSD) Study, researchers also identified significant differences in DTN time when comparing regular hours (Monday–Friday, 7:01 a.m.−5:59 p.m.) to off hours (Monday–Friday 6 p.m.−7 a.m., all day Saturday, all day Sunday, and government holidays) (5). They observed off-hours DTN time to be greater than work hours DTN time likely secondary to increased difficulty obtaining collateral information as well as fewer stroke specialists and staff at night, during weekends, and on holidays. They also found statistically significant reduced DTN time if the patient was brought in by ambulance (5). Walk-in patients do not have the luxury of giving the ED advance notice.

This mirrors our data, which showed an increased DTN time if the patient walked into the ED as opposed to arriving by ambulance. Advanced notice allows the ED personnel and stroke team to prioritize their tasks in the anticipation of giving their undivided attention to a stroke patient. Another explanation for improved DTN time in patients brought by EMS is their ability to correctly identify patients with possible stroke, thereby expediting their care. Many prehospital stroke scales exist, with a sensitivity as high as 85% and a specificity as high as 97% (10). A county-wide EMS stroke protocol found a Los Angeles Motor Score of >4 has excellent predictive validity for identifying patients with large vessel occlusions, resulting in accurate transport decisions to comprehensive stroke centers where thrombolytics are administered promptly, often while the patient is on the way to the angiography suite (11–13). Partnering with one's EMS agency results in more expeditious care, including shorter door-to-physician and door-to-CT time, and greater odds of receiving thrombolysis (14).

Finally, there may be some lingering bias between walk-in patients and EMS patients, namely that patients brought in by ambulance are sicker. Anchoring bias is also very prevalent. Whichever first working “diagnosis” is made is the one that sticks. Therefore, for example, if EMS deems the patient to be a “stroke alert” then stroke protocol in the ED proceeds regardless of any downstream thoughts of alternative diagnoses. Similarly, if EMS brings in a decreased level of consciousness patient but labels them an overdose rather than a stroke, then the natural tendency is for the ED to proceed down the overdose protocol initially. We actually did see this exact scenario in our cohort. This results in a delayed door-to-needle time when the stroke is ultimately found to be the cause.

Our study did not survey the perspectives of physicians and other medical staff, but it would be an aspect to explore in future. While we can only speculate the contributing factors to the data seen in this study, other areas in which future research is needed are the assessments of demographics in those who received thrombolytics, as well as the degree of sickness upon presentation. If a patient must be stabilized prior to undergoing a CT scan and/or administering thrombolytics because of blood pressure, airway compromise, or other condition, this will increase DTN time.

## Limitations

This is a single-center study performed in the Southeastern USA, and thus limited by the population and protocols of this region. Nevertheless, our findings underscore the importance of 24/7 stroke team presence and the importance of our EMS partners.

## Conclusion

Our prospective observational study in a real-world community emergency department showed that arrival during the night shift and as a walk-in rather than *via* ambulance resulted in a significantly longer DTN time. Contributing factors to these findings include fewer staff at night and a delay in identifying patients with acute stroke. Ongoing assessment of local factors that prolong DTN times is an important way to expedite thrombolytics for our patients and address quality metrics.

## Data availability statement

The raw data supporting the conclusions of this article will be made available by the authors, without undue reservation.

## Ethics statement

The studies involving human participants were reviewed and approved by University of Central Florida Institutional Review Board. Written informed consent for participation was not required for this study in accordance with the national legislation and the institutional requirements.

## Author contributions

LG conceived the study and wrote the manuscript. PB, MK, and AG supervised the conduct of the study. TS provided the statistical analyses. AH and JT collected the data. All authors contributed to the revising the original draft manuscript, read the final version of the paper, and have approved it for submission.

## References

[1] Man S Xian Y Holmes DN Matsouaka RA Saver JL Smith EE. Association between thrombolytic door-to-needle time and 1-year mortality and readmission in patients with acute ischemic stroke. JAMA. (2020) 323:2170–84. 10.1001/jama.2020.5697 32484532 PMC7267850

[2] Powers W Rabinstein A Ackerson T Adeoye OM Bambakidis NC Becker K. 2018 guidelines for the early management of patients with acute ischemic stroke: a guideline for healthcare professionals from the American Heart Association/American Stroke Association. Stroke. (2018) 49:e46–99. 10.1016/j.jvs.2018.04.007 29367334

[3] McDermott M. 2018 AHA/ASA Stroke Early Management Guidelines. Washington, DC: American College of Cardiology (2018).

[4] Fonarow GC Zhao X Smith EE Saver JL Reeves MJ Bhatt DL. Door-to-needle times for tissue plasminogen activator administration and clinical outcomes in acute ischemic stroke before and after a quality improvement initiative. JAMA. (2014) 311:1632–40. 10.1001/jama.2014.3203 24756513

[5] Oluwole SA Wang K Dong C Ciliberti-Vargas MA Gutierrez CM Yi L. Disparities and trends in door-to-needle time: the FL-PR CReSD Study (Florida-Puerto Rico Collaboration to Reduce Stroke Disparities). Stroke. (2017) 48:2192–7. 10.1161/STROKEAHA.116.016183 28706119 PMC5639478

[6] Ekundayo OJ Saver JL Fonarow GC Schwamm LH Xian Y Zhao X. Patterns of emergency medical services use and its association with timely stroke treatment: findings from get with the guidelines–stroke. Circ Cardiovasc Qual Outcomes. (2013) 6:262–9. 10.1161/CIRCOUTCOMES.113.000089 23633218

[7] Mochari-Greenberger H Xian Y Hellkamp AS Schulte PJ Bhatt DL Fonarow GC. Racial/ethnic and sex differences in emergency medical services transport among hospitalized US Stroke patients: analysis of the national get with the guidelines-stroke registry. J Am Heart Assoc. (2015) 4:e002099. 10.1161/JAHA.115.002099 26268882 PMC4599467

[8] Kamal N Shand E Swanson R Hill MD Jeerakathil T Imoukhuede O. Reducing door-to-needle times for ischaemic stroke to a median of 30 minutes at a community hospital. Can J Neurol Sci. (2019) 46:51–6. 10.1017/cjn.2018.368 30516454

[9] Köhrmann M Schellinger PD Breuer L Dohrn M Kuramatsu JB Blinzler C. Avoiding in hospital delays and eliminating the three-hour effect in thrombolysis for stroke. Int J Stroke. (2011) 6:493–7. 10.1111/j.1747-4949.2011.00585.x 21609415

[10] Stead TG Banerjee P Ganti L. Real-world field performance of the Los Angeles motor scale as a large vessel occlusion screen: a prospective muticentre study. Cerebrovasc Dis. (2021). 10.1159/000516116 34004604

[11] Abboud ME Band R Jia J Pajerowski W David G Guo M. Recognition of stroke by ems is associated with improvement in emergency department quality measures. Prehosp Emerg Care. (2016) 20:729–36. 10.1080/10903127.2016.1182602 27246289

[12] Stead TG Ganti L Banerjee P. Large vessel occlusion identification through prehospital administration of stroke scales: a county-wide emergency medical services prospective research protocol. Cureus. (2019) 11:e5931. 10.7759/cureus.5931 31788388 PMC6858264

[13] Ganti L Oostema JA. How accurate are the stroke severity scales for identifying large vessel occlusions? Ann Emerg Med. (2020) 75:494–6. 10.1016/j.annemergmed.2019.07.022 31561996

[14] Ganti L Banerjee P Falco W. Partnering with your EMS agency to improve stroke outcomes. ACEP E-QUAL webinar. Available online at: https://vimeo.com/465118080/4349c3db4e (accessed January 20, 2023).

